# Active Site Gate Dynamics Modulate the Catalytic Activity of the Ubiquitination Enzyme E2-25K

**DOI:** 10.1038/s41598-018-25476-8

**Published:** 2018-05-03

**Authors:** Manoj K. Rout, Brian L. Lee, Aiyang Lin, Wei Xiao, Leo Spyracopoulos

**Affiliations:** 1 Department of Biochemistry, Faculty of Medicine and Dentistry, University of Alberta, Edmonton, Alberta T6G 2H7 Canada; 2 Department of Microbiology and Immunology, University of Saskatchewan, Saskatoon, Saskatchewan S7N 5E5 Canada

## Abstract

The ubiquitin proteasome system (UPS) signals for degradation of proteins through attachment of K48-linked polyubiquitin chains, or alterations in protein-protein recognition through attachment of K63-linked chains. Target proteins are ubiquitinated in three sequential chemical steps by a three-component enzyme system. Ubiquitination, or E2 enzymes, catalyze the central step by facilitating reaction of a target protein lysine with the C-terminus of Ub that is attached to the active site cysteine of the E2 through a thioester bond. E2 reactivity is modulated by dynamics of an active site gate, whose central residue packs against the active site cysteine in a closed conformation. Interestingly, for the E2 Ubc13, which specifically catalyzes K63-linked ubiquitination, the central gate residue adopts an open conformation. We set out to determine if active site gate dynamics play a role in catalysis for E2-25K, which adopts the canonical, closed gate conformation, and which selectively synthesizes K48-linked ubiquitin chains. Gate dynamics were characterized using mutagenesis of key residues, combined with enzyme kinetics measurements, and main chain NMR relaxation. The experimental data were interpreted with all atom MD simulations. The data indicate that active site gate opening and closing rates for E2-25K are precisely balanced.

## Introduction

Neurodegenerative diseases such as Alzheimer’s (AD), Parkinson’s (PD), Huntington’s (HD) disease and amyotrophic lateral sclerosis (ALS) share a common mechanism of pathogenesis: the build-up of misfolded toxic proteins and protein aggregates in brain cells. This is due in part to impaired clearance mechanisms, whose efficiency decreases with age^1^. A growing body of evidence points at protein quality control by the ubiquitin proteasome system (UPS), which tags proteins with ubiquitin (Ub) for removal from the cell, as an important player in many neurodegenerative disorders^2,3^. The UPS system attaches lysine 48-linked (K48) polyubiquitin (polyUb) chains on target proteins, which destines them for removal from cells^4^. Cell cycle control^5^, trafficking of cell surface receptors^6^, and activation of transcription by NF-κB^7^, are regulated by attachment of K48-polyUb chains to target proteins. This post-translational modification signals for degradation of the protein by the proteasome^8^. Alternatively, covalent attachment of K63-polyUb chains to proteins initiates signaling cascades that lead to DNA repair^9–11^, altered gene expression^12^, or autophagy^13^.

Mutations within the enzymes and proteins of the UPS contribute to the pathogenesis of disease states, including neurodegeneration, cancer, and viral infection^14–17^. The UPS attaches Ub to target proteins in three steps, starting with attachment to a Ub-activating enzyme, or (E1), a transfer to a Ub-conjugating enzyme (E2), with final attachment of Ub to a target by a Ub-ligase (E3)^14^. The UPS regulates biochemical processes using one E1 enzyme, ~40 E2s, and hundreds of E3s, which provide recognition of targets^18^. The Ub signal has a diverse architecture, variants include attachment of single, or multiple Ub molecules to different sites on a protein, or polyubiquitination through single (homotypic), or variable types (heterotypic) of Ub-Ub peptide bonds^19^. Seven types of homotypic polyUb chains are formed through seven surface lysines on Ub^19,20^; the most highly studied chains are those linked through K48 or K63. An understanding of the remaining chains is less well developed, though generally, it is likely that different chains are involved in different physiological processes. The topological variety for the Ub signal likely gives organisms a biological advantage for achieving specificity in different signaling cascades and biological functions.

The E2 enzyme E2-25K is notable for its potential involvement in AD^21^ and HD^22,23^. Paradoxically, E2-25K catalytic activity has been shown to increase cell death in AD and HD models, potentially through proteasome inhibition^21^. In HD models, misfolded mutant huntingtin (mHTT) protein is tagged with K48-polyUb and removed by the UPS, or alternatively, tagged with K63 polyUb chains for degradation through the autophagic pathway^24^. Disruption of these processes, or an imbalance between K48/K63 ubiquitination can lead to accumulation of toxic mHTT^25^. In this regard, inhibition of E2-25K has been shown to decrease the build-up of toxic mHTT fragments^23^. The mechanism is currently unknown but may be a result of the formation of unanchored polyUb chains that bear UBB+, a frameshift Ub mutant that is known to be a potent inhibitor of the proteasome^21^.

Generally, these observations indicate that E2 enzymes possess precise reaction rates that are critical for maintaining cellular homeostasis. E2 enzymes catalyze the central step in the UPS, and achieve a 10^6^-fold acceleration for the second step of the UPS, or the aminolysis of the Ub thioester bond (nucleophilic attack of lysine on the E2 ~ Ub thioester bond)^17,26^. E2s orchestrate many functions, including shifting from initiation of polyUb chain synthesis to chain elongation, selection of Ub lysines for chain links, and precise timing of chain release to control chain length^27^. Ultimately, E2-dependent ubiquitination of specific proteins is regulated by weak binding of substrates and E3 Ub ligases^16,28–30^. Enzyme kinetic studies have revealed that specificity for E2 enzymes is achieved through variability in chemical mechanism^16,17,26^. Modest differences in the K48-polyUb chain building rate by the SCF Ub ligases gives rise to a large difference in the ability to signal for protein degradation^16^. Additionally, modest decreases in the rate of K63-polyUb chain synthesis by the K63-specific E2 Ubc13 can give rise to defects in the ability of cells to respond to DNA damage^26^. From a mechanistic perspective, the reaction rate of Ubc13 is regulated by the closing and opening dynamics of an active site gate, which connects the α2 and α3 helices, and buttresses the active site cysteine^26^. The gate fluctuations between the open and closed states are precisely balanced; small deviations in gating impair the ability of cells to respond to DNA damage^17,26^.

**Figure 1:**
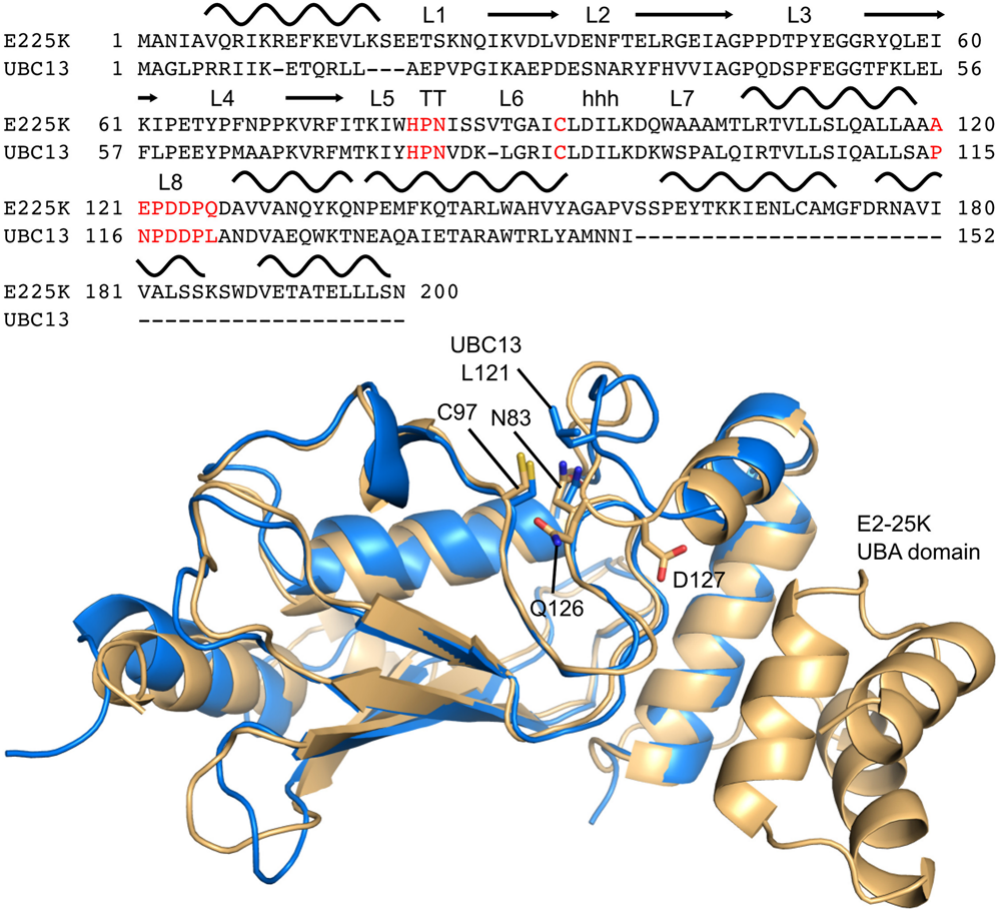
Sequence (top) and structure (bottom) alignments for E2-25K and Ubc13. Secondary structures and loop numbers are shown for the sequence alignment, with the HPN motif, catalytic cysteine, and the active site DPNPxxPL loop motif highlighted in red. The structures of E2-25K (gold) and Ubc13 (blue) correspond to PDB IDs 3K9O and 1J7D, respectively, key residues and the E2-25K UBA domain are indicated.

Interestingly, the active site loop from free Ubc13 adopts a conformation that is not typically observed for E2 active site gates (Fig. 1)^31^. The typical sequence for the canonical E2 loop is DPNPxxPL, where x is a small polar or charged residue^31^. Two conformations have been observed for the active site loop, with the leucine residue exposed in Ubc13^32^, but packed against the core fold in canonical E2s, maintaining the loop in a “closed”, catalytically active conformation^30^. Ultimately, Ubc13 adopts a closed conformation to facilitate catalysis, as observed for the structure of the Mms2-Ubc13 holoenzyme with covalently attached “donor” Ub^33,34^. Mutation of the loop leucine in Ubc13 (L121) to a series of hydrophobic residues of increasing size (G, A, V, I), revealed that it functions to promote solvation substitution at the active site^35^, in order to enhance electrostatic stabilization of the transition state^26^. Furthermore, mutation of the key hinge residue (A122) within the loop to glycine, revealed the key role of dynamics in the catalytic activity of Ubc13^26^.

The active site loop in E2-25K adopts the canonical closed conformation^36^, however, the typical leucine residue within the loop is glutamine (Fig. 1). To understand the role of active site gate residues in E2-25K catalysis, we mutated the central loop glutamine to leucine, isoleucine, alanine, valine, and glycine, and conducted enzyme kinetics assays. To assess the role of loop dynamics on enzyme activity, we mutated the hinge residue D127 to glycine; this is the equivalent residue to A122 in Ubc13. In addition to enzyme kinetics measurements, the impact of mutations on the structural and dynamics properties of E2-25K were studied using NMR backbone resonance chemical shift changes and ^15^N NMR relaxation. Atomic views of the loop motions and estimates for the barrier heights of gate opening for E2-25K as well as the Q126L and D127G mutants, were obtained from all atom molecular dynamics simulations. This combined approach has revealed key mechanistic and dynamic differences between canonical and non-canonical active site loops in E2 enzymes.

## Results

### NMR derived secondary structures for active site E2-25K gate mutants are similar to wildtype

**Figure 2:**
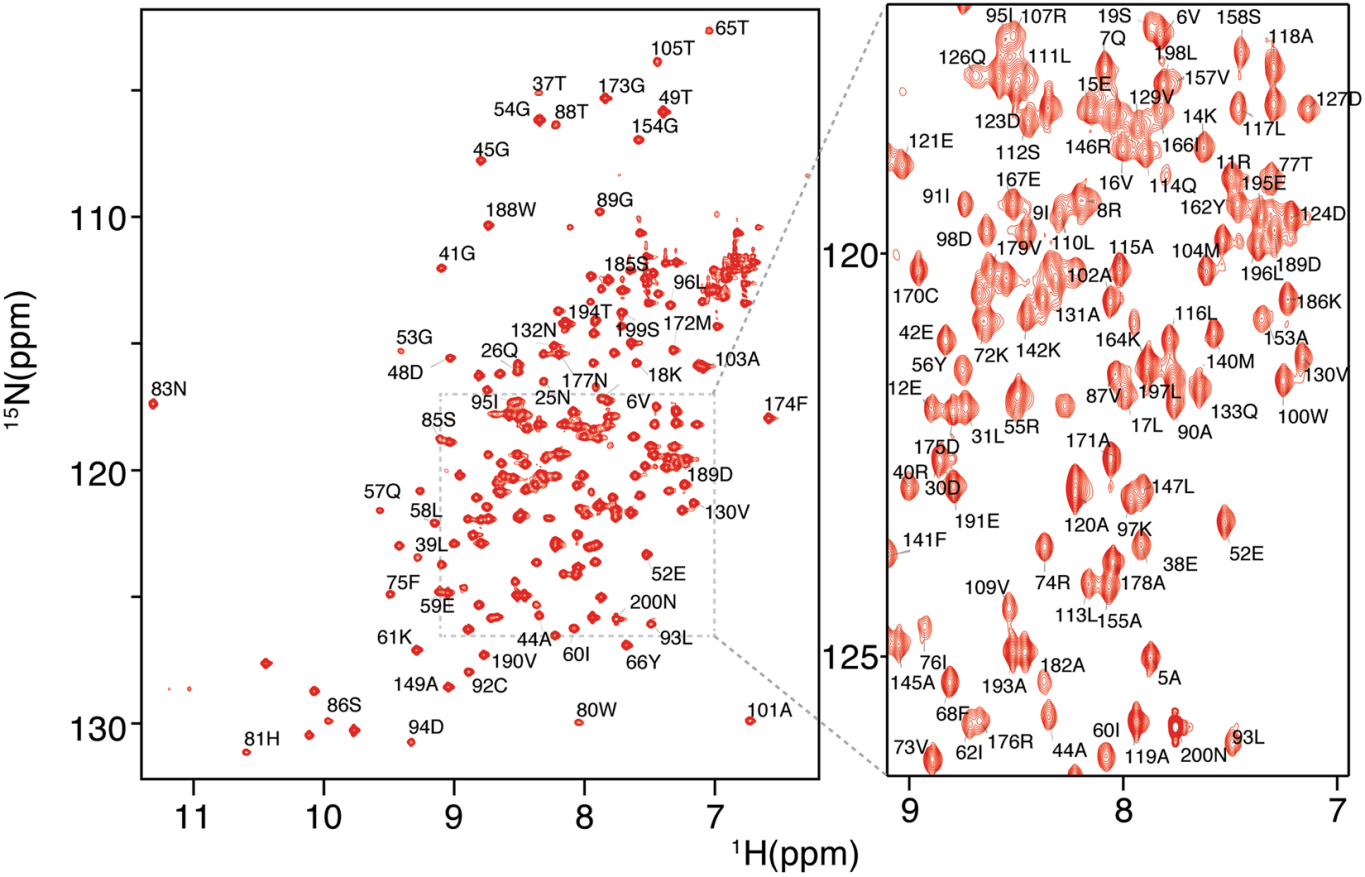
^15^N-^1^H^N^ HSQC spectrum for E2-25K.

**Figure 3:**
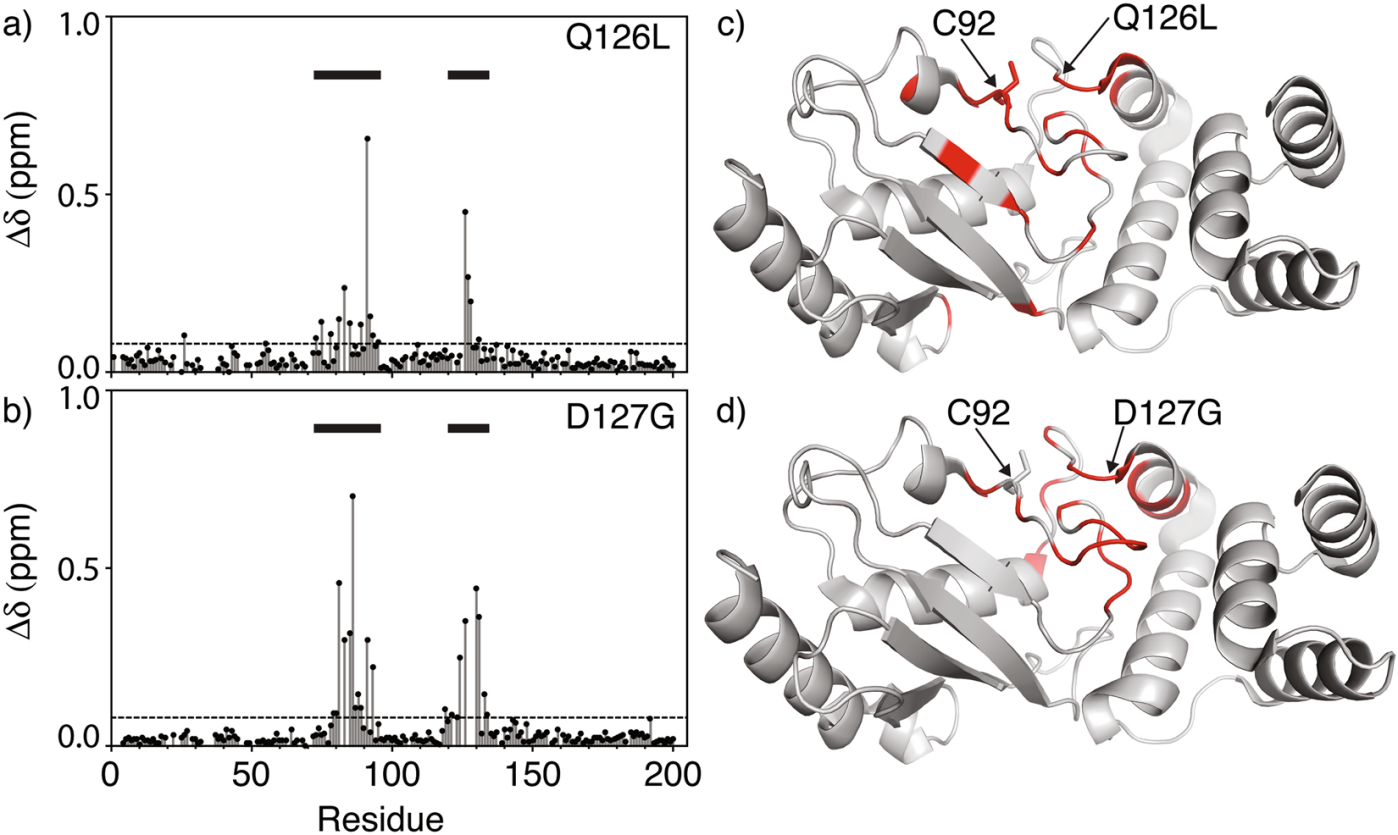
^1^H^N^-^15^N chemical shift perturbations (Δδ) for E2-25K Q126L and D127G active site loop mutations. The active site, residues 75–95, and the active site loop, residues 120–135, are indicated with horizontal bars. Residues with significant chemical shift changes (>Δδ + 1σ) are shown on the main chain structure (red) for Q126L (**c**) and D127G (**d**).

The ^1^H-^15^N HSQC NMR spectrum for wildtype E2-25K is shown in Fig. 2. Chemical shift perturbations (Δδ) indicate that the majority of residues are unaffected by the Q126L and D127G substitutions, apart from residues immediately adjacent to the active site Cys (residues 75–95), and the active site loop (residues 120–135), (Fig. 3). For the D127G mutant, the chemical shift perturbations within the active site loop arise from changes in local environment resulting from the mutations, whereas the larger Δδ values in the active site (residues 75–95) reflect the close coupling of the active site to the buttressing active site loop (residues 120–135). For Q126L, there are fewer changes in chemical shift for the loop region, and changes are less dispersed across the loop in comparison to D127G. The secondary structures of E2-25K and the Q126L/D127G mutants were determined from main chain ^15^N, ^1^H^N^, CO and C_α_ chemical shifts using the CSI 3.0 program (Supplementary Fig. S1). The secondary structures for Q126L and D127G are similar to those for wild type, indicating that the structural and dynamic changes for these mutants are localized to the active site and gating loop.

### NMR monitored titration indicates that the interaction of Ub with E2-25K occurs with weak affinity and fast kinetics

**Figure 4:**
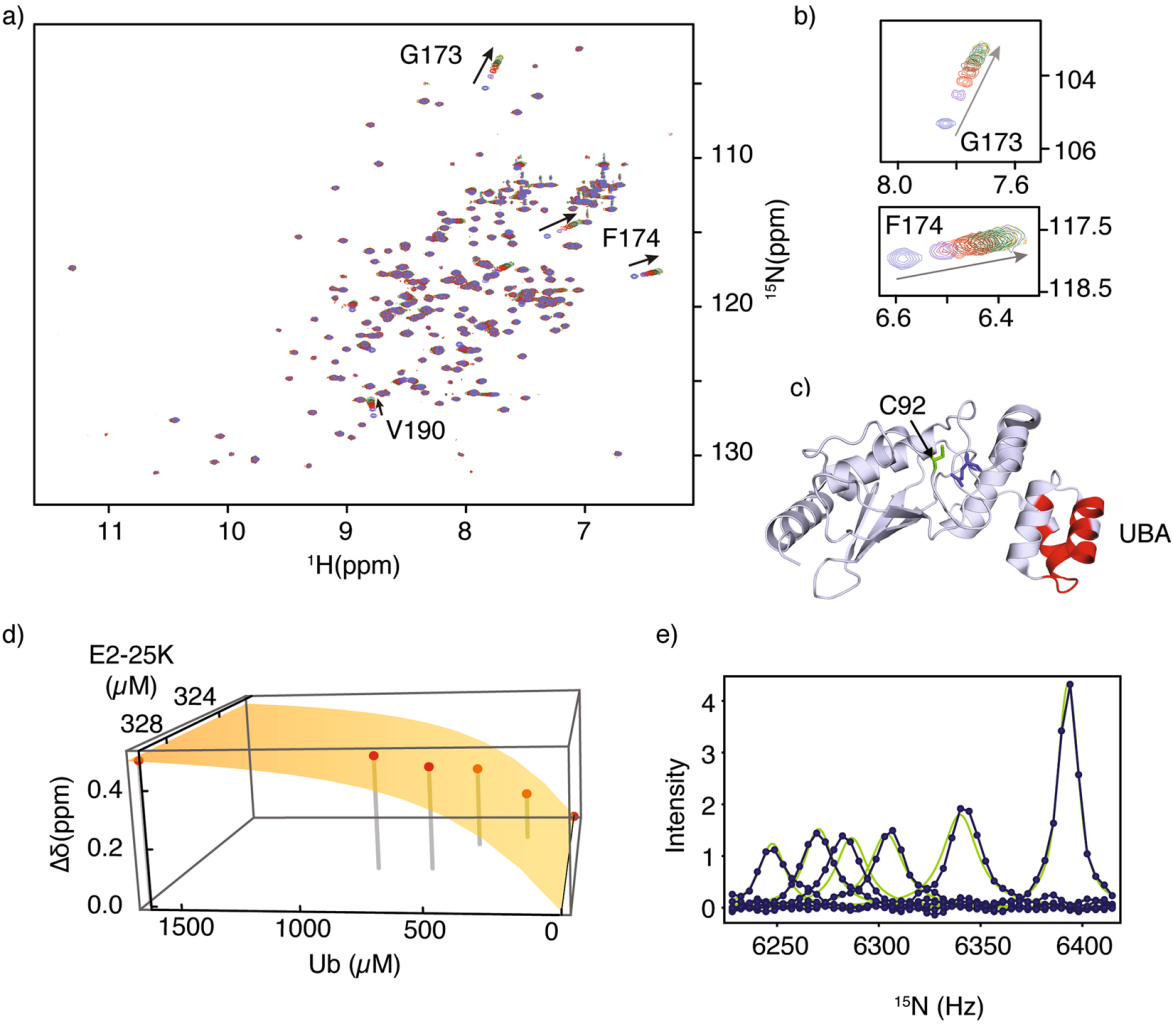
(**a**) Superposition of E2-25K ^1^H-^15^N HSQC NMR spectra upon titration with Ub, arrows indicate chemical shift changes with increasing Ub concentration. (**b**) Expansion of the spectra around G173/F174, residues experiencing significant chemical shift changes upon interaction with Ub, arrows indicate increasing Ub concentration. (**c**) Significant chemical shift perturbations (>Δδ + 1σ, red) upon Ub binding, mapped onto the main chain structure of E2-25K. The active site C92 (green) and gate residue Q126 (blue) are shown in the stick representation. (**d**) Chemical shift changes for G173 upon titration with Ub fit to a 1:1 binding isotherm, experimental shifts are shown as points, and the best fit as a surface. (**e**) ^15^N lineshape analysis for residue G173 with experimental data shown as points (blue), and the best fits shown as green lines.

The UBA domain from E2-25K, in conjunction with the core UBC domain, functions to position an “acceptor” Ub to facilitate catalysis of the synthesis of K48-linked Ub chains. We monitored the interaction of Ub with E2-25K using ^1^H^N^-^15^N HSQC NMR spectroscopy (Fig. 4a). The largest chemical shift perturbations (Δδ) include residues G173, F174, and V190 within the UBA domain (Fig. 4b), accompanied by significant Δδ values that exceed the average chemical shift by 1 σ (Fig. 4c). From the titration, Δδ values yield an average dissociation constant (*K*_*D*_) of 206 ± 30 µM for 1:1 protein-protein binding (Fig. 4d). It has previously been shown that binding of Ub by the human E2-25K UBA domain alone is extremely weak, *K*_*D*_ ~ 1.2 ± 0.3 mM^37^. The *K*_*D*_ value we measure for Ub binding to intact human E2-25K is consistent with the *K*_*D*_ of 228 ± 60 µM for Ub binding to yeast E2-25K, measured using NMR methods^36,38^, and the apparent *K*_*D*_ (or *K*_*M*_ value) for Ub binding of ~350 µM derived from K48-Ub_2_ chain synthesis assays^39^. These dissociation constants for yeast and human E2-25K indicate that both the UBC and UBA domains interact with ubiquitin, demonstrating the fundamental principle of catalysis by proximity and orientation for E2-25K. Using spectra from our NMR titration, we also conducted ^15^N NMR lineshape analysis for the main chain amide of G173, to determine rate constants for the interaction with *k*_*on*_ = 2.9 × 10^7^ M^−1^ s^−1^ and *k*_*off*_ = 5886 s^−1^ (Fig. 4e). These values were subsequently used in the rate laws describing the kinetics of enzyme assays for synthesis of K48-Ub_2_ chains by E2-25K, described in more detail below, and within the Methods.

### Conjugation of Ub to E2-25K Q126L by E1 enzyme is substantially enhanced compared to wildtype

**Figure 5:**
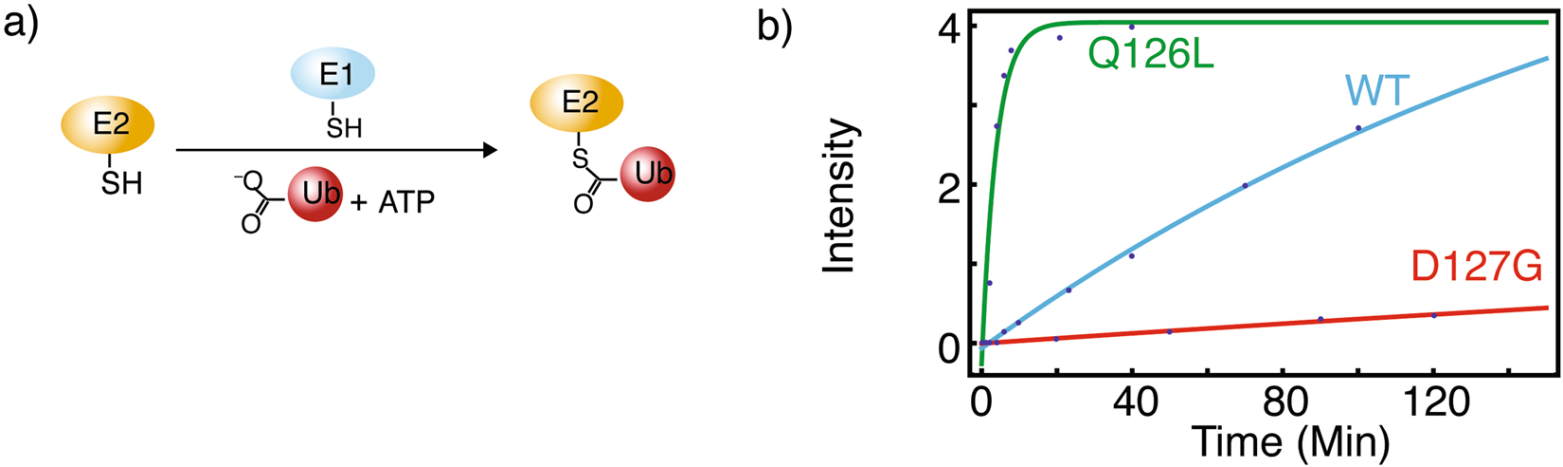
Schematic presentation of ATP-dependent E2-25K thioester formation catalyzed by E1 enzyme (**a**). Fluorescence intensities for thioester synthesis are shown as blue dots, with best fits shown as lines (**b**).

The first step of ubiquitination cascade is the E1-catalyzed, ATP-dependent covalent attachment of the active site cysteine of an E2 with the C-terminal carboxyl of Ub. Under conditions where the concentrations of E2 are the same, accounting for E1 concentrations gives apparent rate constants (*k*_*app*_ = *k*_obs_/[E1]) for conjugation of Ub to E2-25K are 0.041 ± 0.002, 0.0075 ± 0.0003, and 1.8 ± 0.3 (×10^6^ M^−1^ min^−1^) for wildtype, D127G, and Q126L (Figs. 5a,b and S2). Interestingly, the value of *k*_*app*_ for Q126L comparable to the previously measured value of 1.4 ± 0.1 (×10^6^ M^−1^ min^−1^) for wildtype Ubc13^26^. The competing thioester hydrolysis reaction is substantially slower and does not impede aminolysis, with hydrolysis rates of ~0.001 min^−1^, as previously observed for Ubc13^26^.

### Aminolysis of E2-25K Q126L thioester is substantially enhanced compared to wildtype

**Figure 6:**
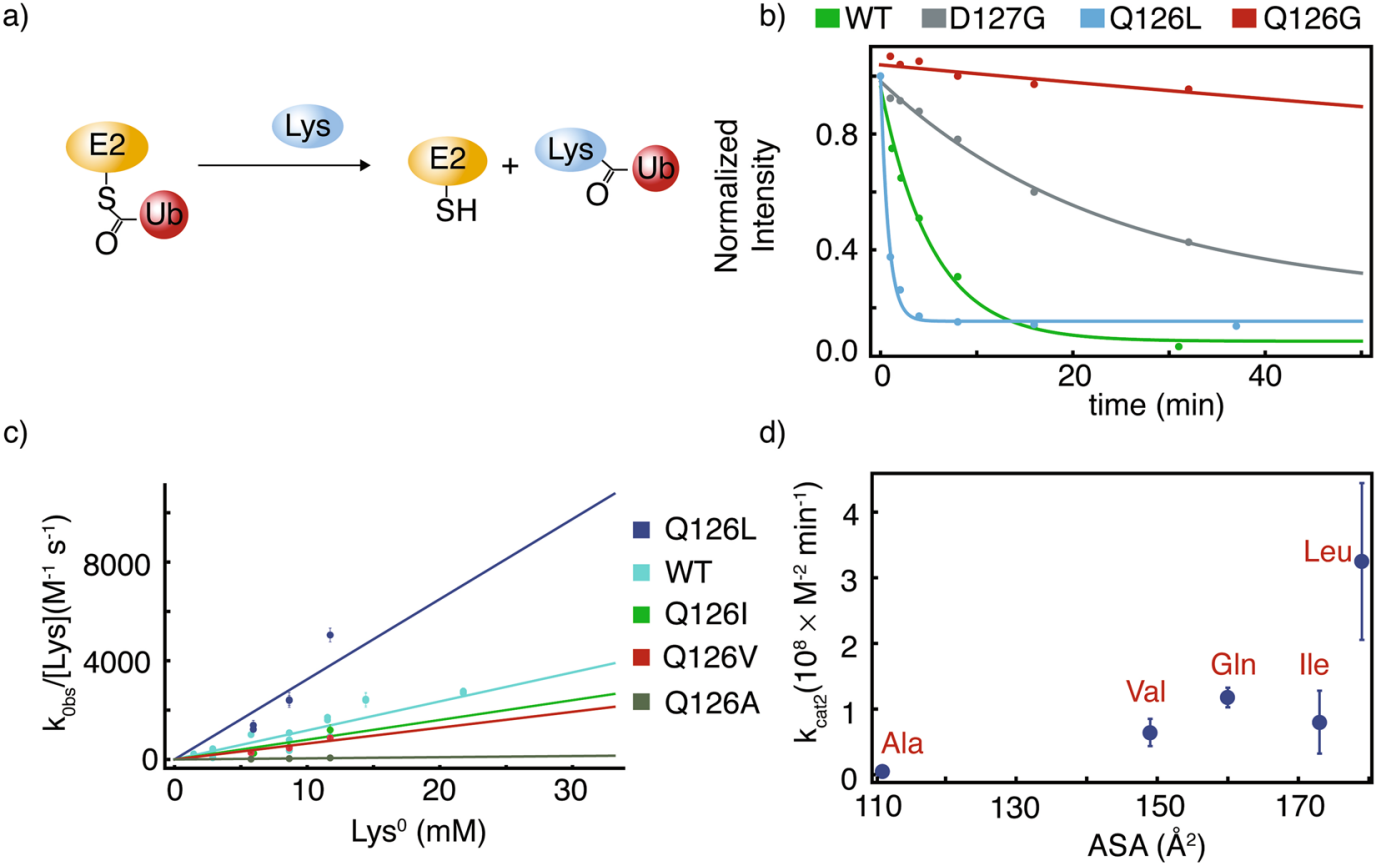
Aminolysis of E2-25K thioester by lysine. For the general reaction (**a**), the kinetics of thioester aminolysis by 30 mM lysine are shown in (**b**), where apparent rates are derived from exponential fits of the data (lines). Second order rate constants (*k*_*cat2*_) derived from the concentration dependence of aminolysis kinetics by neutral lysine are shown in (**c**), and plotted as a function of side chain accessible surface area for position 126 of wildtype E2-25K wildtype (Q), and Q126L, Q126I, Q126V, and Q126A mutants.

The aminolysis of E2 ~ Ub thioester by the nucleophile L-lysine provides a straightforward reaction to facilitate comparison of active site gate mutants^26,40^ (Fig. 6a). The observed catalytic rates of aminolysis (*k*_*obs*_) using 30 mM lysine were 0.19 ± 0.03, 0.05 ± 0.01, and 1.3 ± 0.1 min^−1^ for wildtype E2-25K, and the D127G and Q126L mutants, respectively (Fig. 6b). To understand the role of Q126 in solvation substitution at the active site, we mutated Q126 to the series A, V, I, L, and measured the dependence of aminolysis on lysine concentration. This facilitated determination of the second order rate constant *k*_*cat2*_, which ranges from 0.05–1 (×10^8^ M^−2^ s^−1^) (Fig. 6c). There is an approximately linear increase in *k*_*cat2*_ for the mutants Q126A, Q126V, and Q126I with respect to average accessible surface area (ASA) of the side chain (Fig. 6d). Whilst the ASA value of leucine is only marginally larger compared to isoleucine, the *k*_*cat2*_ value is substantially larger. This result is consistent with kinetic studies using the yeast homolog of E2-25K, Ubc1, for which the mutant Q122L has an increased rate for ubiquitination of sea urchin cyclin B substrate, in comparison to wild type Ubc1^39^. The *k*_*cat2*_ value for lysine aminolysis catalyzed by wildtype E2-25K is fourfold greater than that previously measured for Ubc13^26^, suggesting mechanistic differences between different E2 enzymes.

### E2-25K Q126L and D127G mutants show impaired rates of K48-Ub_2_ synthesis

**Figure 7:**
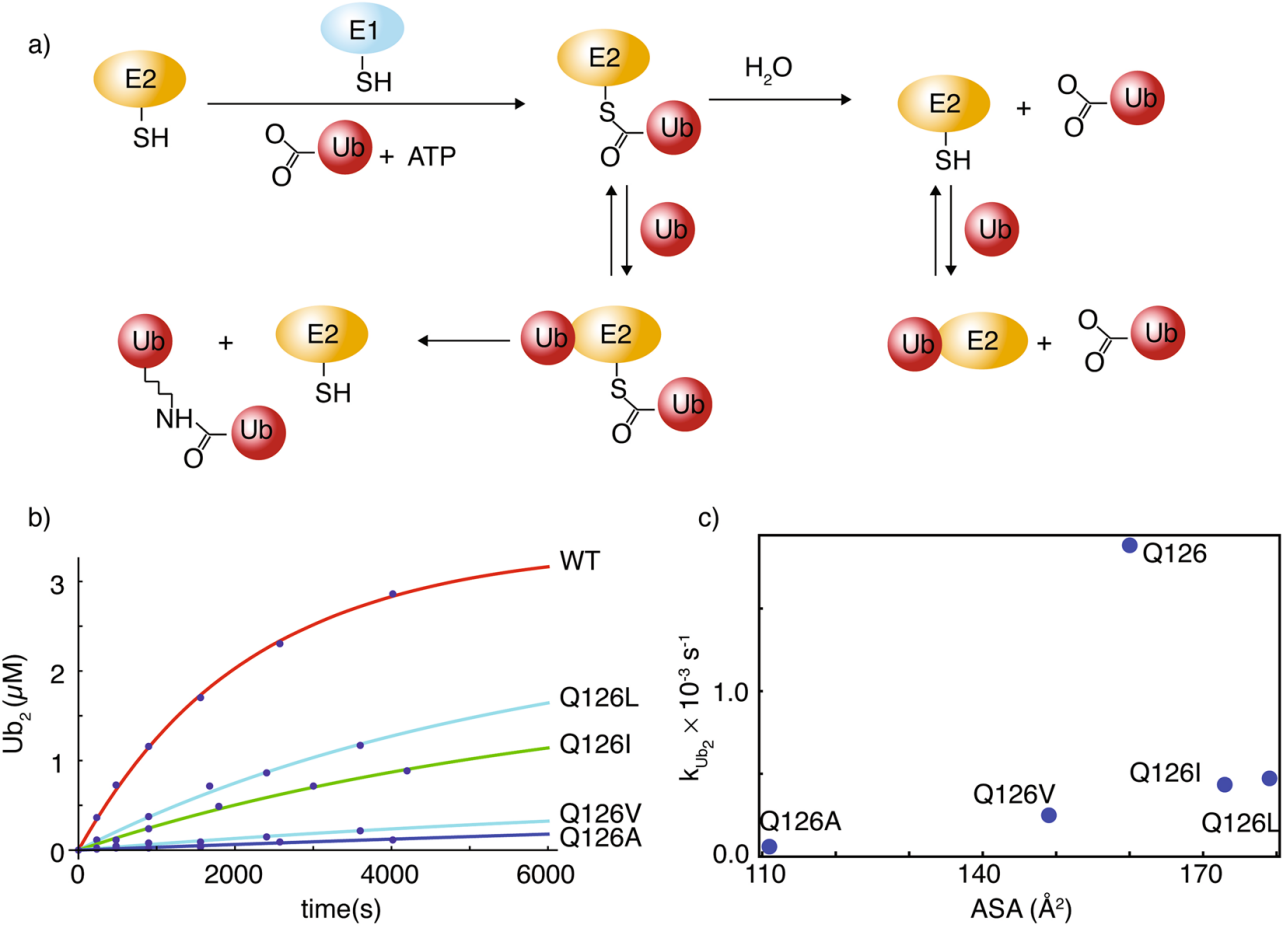
(**a**) Reaction scheme for catalysis of K48-linked Ub_2_ synthesis by E2-25K (**a**). (**b**) Kinetics of K48-linked Ub_2_ chain formation catalyzed by E2-25K and active site gate mutants, with associated fits to yield the *k*_Ub2_ rate constants, plotted as a function of side chain ASA values for gate mutants (**c**).

Binding of Ub by the UBA and UBC domains from E2-25K facilitates catalysis of the synthesis of K48-linked Ub chains in the absence of an E3 Ub ligase^41^. A schematic representation of the kinetic scheme for chain synthesis, which includes concomitant hydrolysis of the thioester bond, is shown in Fig. 7a. To understand the mechanistic aspects of the active site gate for E2-25K, we measured the kinetics of K48-Ub_2_ chain formation (Figs. 7b and S2). The rate constant for K48-Ub_2_ synthesis catalyzed by wildtype E2-25K, *k*_Ub2_, is 0.002 s^−1^, comparable to the value of 0.007 s^−1^ for catalysis of K63-Ub_2_ synthesis by the Mms2/Ubc13 protein complex^17^. Interestingly, *k*_Ub2_ for wildtype is about fourfold greater than for Q126L (Fig. 7c). The gate mutants Q126I, Q126V, and Q126A are partially impaired with respect K48-Ub_2_ chain formation (Fig. 7c). Similar to the results for aminolysis by lysine, the rate of K48-Ub_2_ chain synthesis follows a roughly linear increase as a function of ASA values for the hydrophobic side chains (Fig. 7c). Whilst catalysis of aminolysis is only partially impaired for the E2-25K D127G mutant, K48-Ub_2_ synthesis is completely impaired.

### NMR relaxation measurements indicate increased loop flexibility for E2-25K D127G

^15^N-*R*_1_, -*R*_2_, and NOE NMR relaxation rates were measured at 600 MHz to assess the main chain dynamics for E2-25K and the active site mutants on the pico- to millisecond timescale (Supplementary Fig. S3). The overall rotational correlation times for wildtype, Q126L, and D127G E2-25K were determined from the *R*_2_/*R*_1_ ratio and range from 16–18 ns. In contrast to our previous studies of the active site loop for the E2 Ubc13^26^, resonance overlap and low signal to noise ratios for a number of peaks in the ^1^H-^15^N HSQC NMR spectra hinders comprehensive analysis of loop relaxation rates. However, for the gate mutant D127G, ^15^N-*R*_1_, -*R*_2_, and NOE values at residue 127 indicate increased flexibility in comparison to wildtype (Supplementary Figs. S3 and S4). For the gate mutant Q126L, resonance overlap prevents comprehensive comparison to wildtype, however, ^15^N-*R*_1_, *R*_2_, and NOE values at residues 123 and 126 are not indicative of increased flexibility for the loop.

### MD simulations indicate that the barrier to gate opening for E2-25K D127G decreases compared to wildtype

**Figure 8:**
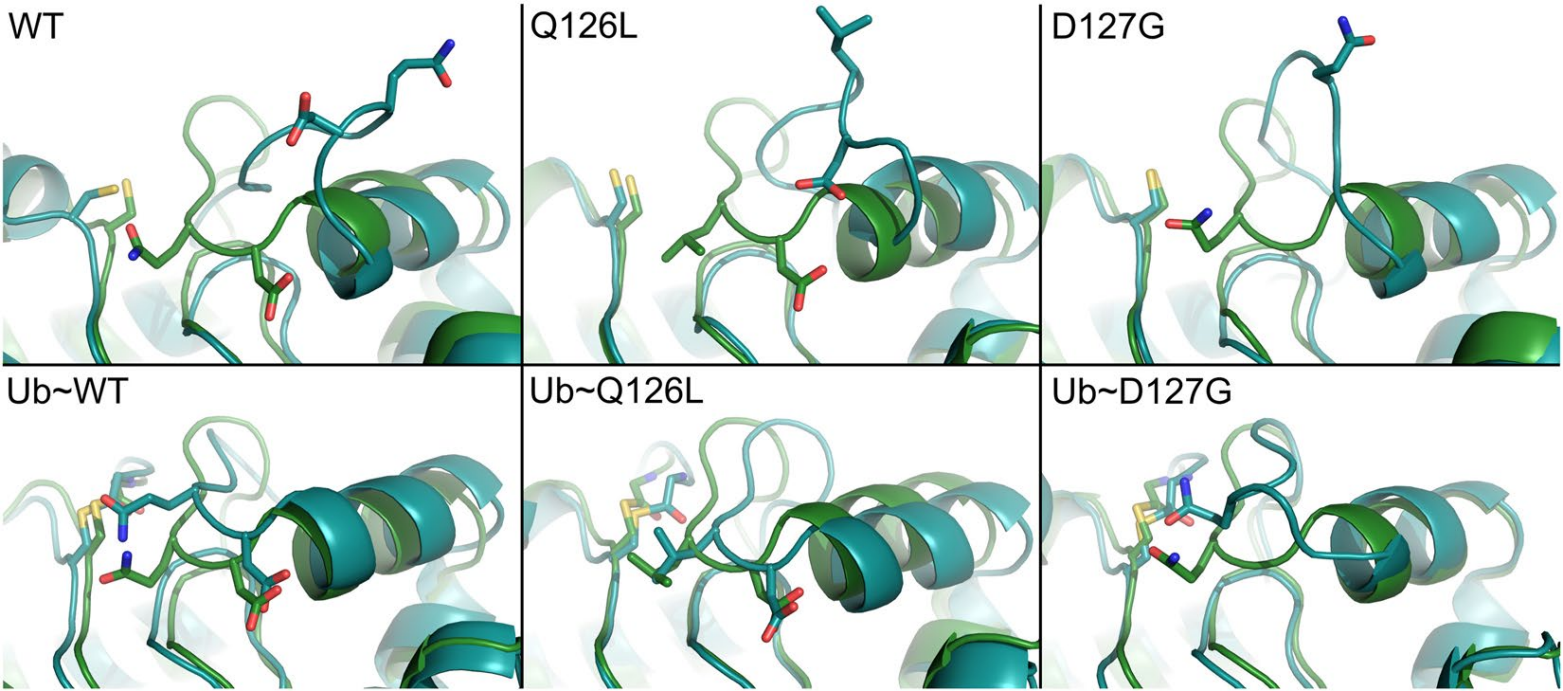
Stochastic gating for the active site loop of E2-25K and various mutants, in the free and Ub-thioester conjugated states. The starting conformations and representative open gate conformations are shown. Gate opening was observed in the free enzyme (top row), and the ubiqutin thioester-linked wildtype and D127G enzymes (bottom row). C92, Q/L126, D127 and G76 from thioester linked Ub are shown in the stick representation.

**Figure 9:**
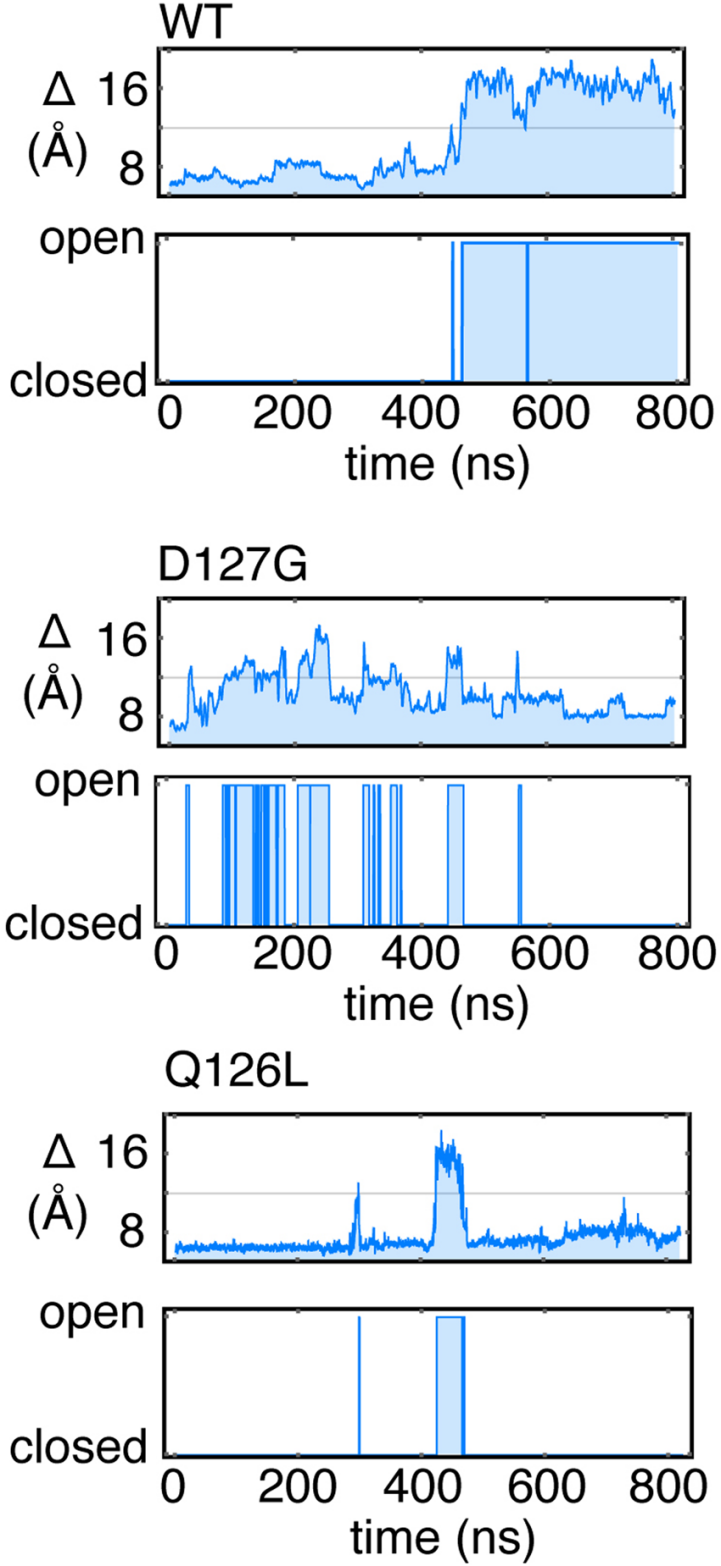
Stochastic gating for the active site loop of E2-25K and various mutants, in the free state derived from MD simulations. The upper panels for each protein indicate the distance between the C_α_ of C92 and the C_α_ of Q126 or Q126L, with the cut-off value between open and closed indicated by a line. The lower panels indicate digitization to a two state Markov chain.

**Figure 10:**
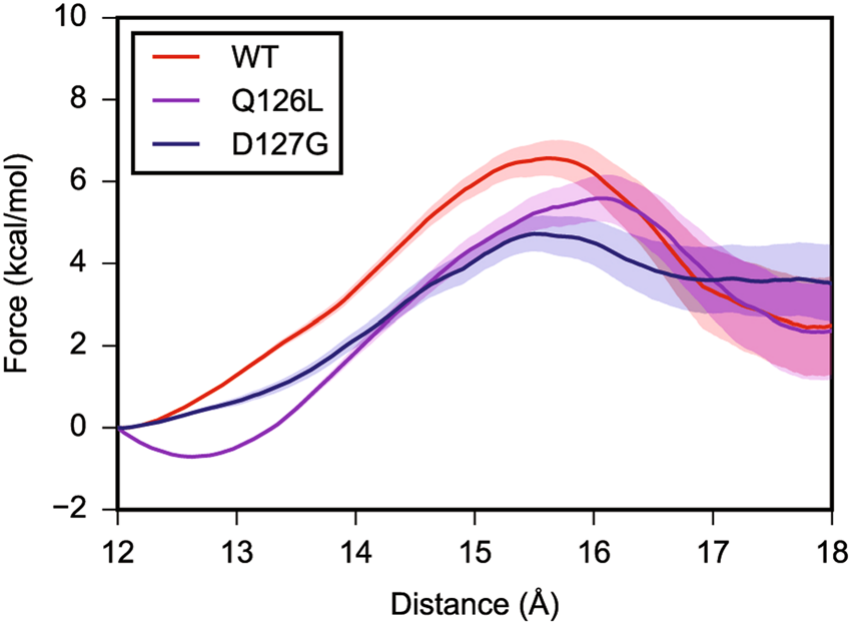
Steered MD simulations for opening of the active site gate of E2-25K and various mutants, in the free state. The *x*-axis indicates the distance between the C_α_ of Q126 or Q126L, and the geometric center of the protein, not including the loop.

The dynamics for E2-25K and the active site gate mutants Q126L and D127G were probed using MD simulations. The active site gate is generally observed to undergo stochastic fluctuations between two states, a closed state for which the distance between the C_α_ atom of C92 within the active site and the C_α_ atom of Q126 at the center of the active site gate is ~6–8 Å, and an open state where the distance increases to ~12–16 Å (Fig. 8). The rates of gate opening are slow over the course of the 400–800 ns simulation times, with only a few transitions to the open state observed for the various proteins; two representative MD runs for each of wildtype, D127G, and Q126L are shown in Fig. 9. The total number of transitions over the total simulation times can be used to provide estimates for the barrier heights of gate opening of 9.2, 8.0, and 7.9 kcal/mol for wildtype, Q126L, and D127G, respectively, with similar barrier heights for closing. We also determined the barrier heights for opening using steered MD simulations, which gave underestimated values in comparison to counting transitions from the MD runs, but a similar decreasing trend for the barrier to gate opening with values of 6.1, 5.6, and 3.8 kcal/mol for wildtype, Q126L, and D127G, respectively (Fig. 10). For simulations involving wildtype, and D127G E2-25K Ub with the C-terminus of Ub covalently attached to the active site Cys through a thioester bond, and Ub in the “closed” conformation, the rates of gate opening are also slow over the 400–800 ns simulation times, with only a few opening transitions observed, similar to the non-thioester simulations. The total number of open and closed transitions over the course of multiple 400–800 ns simulations gave barrier heights for opening of 7.0 and 7.5 kcal/mol for D127G and wildtype, respectively. The magnitude of opening is ~2–3 Å smaller than the non-thioester enzyme but remains sufficient to allow product release. We did not observe opening of the gate for Q126L ~ Ub in MD simulations. However, given that the experimental rates of catalysis for this mutant are faster than wildtype, release of the C-terminus of Ub from the active site is not rate limiting. In general, the hydrogen bond between the carbonyl oxygen of the E2-25K ~ Ub thioester bond and the side chain amide proton of N83 remained stable throughout the simulations for wildtype, Q126L, and D127G E2-25K. Interestingly, during the MD simulations, H81 from the canonical HPN motif of E2 enzymes was observed to rapidly adopt the side chain conformation that stabilizes a tight turn, rather than interact with the side chain of N83, as previously observed using NMR studies^42^.

^15^N-*R*_1_, -*R*_2_, and NOE NMR relaxation values were calculated from the MD simulations for comparison to experimental values acquired at 600 MHz. Twenty nanosecond windows corresponding to either the closed or open states were taken from ~800 ns trajectories for E2-25K and the Q126L and D127G mutants and used to determine the correlation functions for fluctuations of the main chain amide ^15^N-^1^H^N^ vectors, corresponding to either the open or closed states. The correlation functions were fit to a five-parameter exponential decay function to extract correlation times^43^. These correlation times were subsequently used in a five-parameter spectral density function, along with the experimentally determined correlation times (τ_c_) for overall molecular tumbling of E2-25K and the active site mutants, to calculate ^15^N-*R*_1_, *R*_2_ and NOE relaxation parameters for the open and closed states, as previously described^26,43^. The relaxation rates for a given state were then averaged over the number of windows taken from the trajectory and weighted according to the fraction of closed and open states observed during the MD simulations to produce the final rates (Supplementary Figs. S4 and S5). The MD derived relaxation rates indicate that pico to nanosecond main chain dynamics for residues 123–127 of the gating loop from wildtype and Q126L E2-25K are similar, whereas the loop is more flexible at the hinge position 127 for the D127G mutant, evident through decreased MD-derived ^15^N-*R*_1_, *R*_1_, and NOE values. Detailed comparison between MD-derived ^15^N-*R*_1_, *R*_1_, and NOE values and experimental values is hampered as a result of resonance overlap and low signal to noise ratios for the NMR data. However, experimental and MD-derived ^15^N-*R*_2_ and NOE values indicate increased flexibility at hinge residue 127 for the D127G mutant (Supplementary Figs. S4 and S5).

## Discussion

The selective catalysis of covalent isopeptide bonds between Ub and a multitude of target proteins is managed by the three-component ubiquitination enzyme cascade^20^. E2 enzymes catalyze the central step of ubiquitination, linking activation of the C-terminus of Ub with final attachment to target lysines with accessory E3 Ub ligases^27,44^. E2 enzymes possess a loop that buttresses the active site; this loop typically adopts a closed conformation, with a conserved region composed of the sequence PXXPY, where X corresponds to Asp, Asn, Ser, Ala, Glu and His^31^. In general, Y is hydrophobic (Ala or Leu), with the exception of E2-25K and its yeast homolog UBC1, for which this residue is hydrophilic (Q126). For canonical E2 enzymes, including E2-25K, the residue at position Y is generally packed against the active site Cys, in a closed conformation^31^. In contrast, for the E2 enzyme dedicated to catalyzing synthesis of K63-linked chains, Ubc13, L121 at position Y is typically fully exposed to solvent, in an open conformation^32^.

In order for a substrate lysine to be covalently attached to the C-terminus of Ub, the C-terminal tail must insert into the active site cleft of an activated E2, in other words, an E2 with the C-terminus of Ub conjugated to the active site Cys through a thioester bond. Upon entry into the active site, the carbonyl oxygen of the thioester bond forms a hydrogen bond to a buried, conserved Asn residue, and nucleophilic attack of the thioester by the deprotonated lysine amino group can occur^30,45^. Given the close packing between the loop and the active site, entry of the C-terminus of Ub into the active site cleft requires conformational rearrangement and opening of the loop, as previously observed for Ubc13^26^. The opening of the active site cleft, and close packing of thioester-linked Ub on the underside of the E2, is facilitated by E3 Ub ligase accessory proteins, though the mechanistic basis is not clear^29^.

In this study, we examined the role of key active site residues in the catalytic mechanism for E2-25K in the absence of an E3 Ub ligase. Aminolysis of the E2 ~ Ub thioester by L-lysine provides a convenient method to compare the catalytic activity of different E2s, or E2 mutants, in the absence of substrate specific effects^17,40^. Interestingly, mutation of the key glutamine residue at position Y to leucine, results in a substantial increase in the rate of thioester formation by E1, as well as the second order rate constant for aminolysis. The mechanistic importance of this position is highlighted by the roughly linear increase in the second order rate constant of aminolysis with respect to average hydrophobic accessible surface upon systematic mutation of position Y of the loop to A, V, I, and L, combined with the increased rate of aminolysis upon mutation to leucine. A hydrophobic residue at position Y facilitates interactions that stabilize the closed conformation. Furthermore, the linear dependence of the second order rate constant for aminolysis upon systematic mutation of Q126 to increasingly hydrophobic residues indicates that position Y plays a role in solvation substitution. That is, the enhancement of electrostatic interactions within an enzyme active site through exclusion of solvent^35^, as we previously observed for this position in the E2 Ubc13^17^. That these effects are largely static in nature is corroborated by our combined MD and NMR relaxation studies, which indicate that there are no substantive changes in flexibility for the Q126L mutant in comparison to wildtype. It is noteworthy that the Q126L mutant is impaired with respect to catalysis of the synthesis of K48-Ub_2_ chains. This observation indicates that E2-25K is tailored to synthesize K48-linked Ub chains, with Q126 participating in specific interactions with an acceptor Ub bearing the nucleophilic K48 which ultimately reacts with the donor Ub ~ E2-25K thioester bond. Our findings are consistent with kinetic and mutational studies for Ubc1, the yeast homolog of E2-25K, for which position Y is Q122, and is critical for synthesis of K48-linked Ub chains, but which shows significantly enhanced ubiquitination of cyclin B for the Q122L mutant^39^. Thus, position Y is unique amongst active site residues, as it enhances, rather than diminishes catalysis of aminolysis. For example, the active site residues N83 and D124 in E2-25K are conserved in E2s. N83 directly stabilizes the oxyanion, and D124 stabilizes the developing charge on the nucleophilic substrate lysine for the zwitterionic tetrahedral transition state^17,30,46^. Various mutations of these key active site residues reduce catalytic activity^30,46,47^. D123 in E2-25K is expected to position substrate through ionic interactions with R74 from the C-terminus of the donor Ub, as observed for UbcH5A, with mutations that disrupt this interaction reducing catalytic activity^30^.

In addition to electrostatic effects discussed above, reactions with the nucleophile L-lysine facilitate studies of active site gating for E2s. For Ubc13, mutation of the key hinge residue A122 to glycine resulted in a faster rate of gate opening. This increase in the rate of opening, corresponds to a loss in the rate of aminolysis, as catalysis depends upon closing of the gate, to stabilize the zwitterionic charged transition state^26^. For E2-25K, the corresponding hinge residue is D127, and our combined MD and NMR relaxation data indicate that the gate is more flexible for the D127G mutant. Additionally, we estimate that the barrier to gate opening is decreased by ~2 kcal/mol in comparison to wildtype, either by counting transitions during MD simulations, or through steered MD. Using our previously developed kinetic scheme for E2-catalyzed aminolysis^26^, this 2 kcal/mol decrease in barrier height for gate opening translates into an ~7-fold decrease in the rate of aminolysis for D127G at a lysine concentration of 30 mM, a value close to the experimentally determined 4-fold decrease. Interestingly, the D127G mutant is completely impaired with respect to catalysis of synthesis of K48-Ub_2_ chains. This result indicates a role for D127 in substrate binding and positioning, in addition to gating. Thus, as in the case of Ubc13^26^, rates of opening and closing for the E2-25K active site gate appear to be precisely balanced, with an increase in the rate of gate opening resulting in loss of catalytic activity for aminolysis. However, there are some important differences in the gating mechanism of Ubc13 compared to E2-25K. Leucine at position Y within the gate of Ubc13 has only been observed to pack against the active site cysteine and adopt the closed conformation in the presence of the accessory protein Mms2 with Ub conjugated to the active site and bound to the underside of Ubc13^34^, whereas E2-25K generally adopts the catalytically active, closed conformation. Thus, the fourfold larger second order rate constant for lysine aminolysis by E2-25K in comparison to Ubc13^26^, may be a result of E2-25K more effectively adopting the catalytically active closed conformation. It is instructive to compare our results for gating in E2-25K to those of other E2s. Cdc34, Ubc7, and Ube2g2 possess an acidic insertion within loop 7 (L7); a loop that is adjacent to the active site gate (loop 8, or L8). MD simulations have identified open and closed L7 conformations that affect the solvent accessibility of the active site Cys^48,49^. Interactions were observed between L7 and L8, and their dynamics were correlated. Phosphorylation of a serine on the central helix promotes an open conformation of L7 as a result of electrostatic repulsion and is thought to facilitate charging of E2 by E1. The acidic loop may also stabilize E2-Ub interactions. Residue 120 within L8 in human Ube2A, and its yeast homolog Rad6, is generally an acidic residue or a serine which can be phosphorylated. MD simulations indicate that phosphoryation of S120 in Ube2a increases the solvent accessibility of the active site Cys, and that negative charge and residue size at this position are important for coordinating the substrate lysine^50^.

It is interesting that the barrier to gate opening for E2-25K is relatively large, even though the rate constants for aminolysis and K48-linked ubiquitination for wildtype E2-25K are at least 10^6^-fold greater than the uncatalyzed rate constants, as previously observed for Ubc13^17^. This result points to the important dual role of E3 ligases, not only do they provide target specificity for E2-catalyzed ubiquitination, they also provide a relatively small, but biologically crucial increase in the basic E2 rate of catalysis^28^, to ensure accurate target ubiquitination. Structural studies indicate that the RING-type E3 ligases stabilize the close packing of the donor, or thioester-linked Ub on the underside of the E2 in a closed conformation to enhance catalysis^29,30^. However, the relationship between E3-mediated ubiquitination to E2 active site gating and facilitating the closed E2 ~ Ub conformation is currently not well understood. In this regard, MD simulations for the SUMO-specific E2 Ubc9 in complex with SUMO (a Ub-like modifier), the E3 RanBP2, and substrate RanGAP1, indicate that the overall motions of Ubc9, including those of the substrate binding site, and L8, are reduced upon SUMO and E3 binding, with the dynamics around the active site becoming more correlated^51,52^. Interestingly, combined NMR and MD studies of the activation of Ube2g2 by two domains from the E3 gp78, the helix from the G2BR domain and the RING domain, have demonstrated that the E2 becomes more rigid upon binding G2BR and L7 with an acidic insertion becomes more closed. However, on subsequent binding of the RING domain from gp78, the extended L7 acidic loop equilibrates between being partially open and closed, which is believed to enable or facilitate subsequent substrate lysine attack^53^. Our previous study of the E2 enzyme Ubc13^17,26^, and E2-25K in the present work, both in the absence of an E3 Ub ligase, indicate that opening and closing rates are precisely balanced. It will be of interest to determine if E3 ligases promote a more open gate to facilitate entry of the C-terminus of Ub into the E2 active site, or if the mechanism involves only stabilization of the close packing of Ub onto the underside of the E2.

## Methods

### Cloning, protein expression, and purification of E2-25K and active site gate mutants

Human E2-25K was cloned into the pHis-parallel1 vector. Site-directed mutagenesis was performed using a QuikChange mutagenesis kit to obtain the active site gate mutants D127G, Q126G, Q126L, Q126V, Q126A and Q126I. Expression of unlabeled protein was initiated by transforming 50 μL of *Epicurian coli* BL21(DE3)-RIPL electro-competent cells with 100 ng of plasmid DNA, followed by overnight growth on agar plates. A single colony was used to inoculate 50 mL of LB starter culture containing ampicillin, and grown overnight at 37 °C. Subsequently, 5 ml of overnight starter culture were used to inoculate 500 mL of LB containing ampicillin and chlormaphenicol. Protein expression was induced through addition of 0.4 mM 1-thio-β-D-galactopyranoside upon growth of the cell culture to an *A*_600_ of ~0.6, and incubated at 25 °C overnight. For the production of [U-^15^N] and [U-^13^C,^15^N] E2-25K for NMR studies, M9 minimal medium containing (^15^NH_4_)_2_SO_4_ and ^13^C-glucose as the main nitrogen and carbon sources, respectively, was employed as described previously for the protein RAP80^54,55^. Following protein overexpression, cells were harvested by centrifugation, suspended in 100 mL of lysis buffer (500 mM NaCl, 20 mM imidazole, 1 mM DTT, 100 µg/ml DNase I, 10 mM MgSO_4,_ 0.5% protease inhibitor cocktail and lysozyme), and lysed using sonication. The cell lysate was subjected to centrifugation, the supernatant was passed through a 0.22 µm filter, and loaded onto a His-prep FF16/10 column equilibrated with binding buffer (20 mM imidazole, 20 mM sodium phosphate, 500 mM NaCl, 1 mM DTT, pH 7.4). Following binding and washing with seven column volumes of binding buffer, E2-25K was eluted using a gradient of 20–500 mM imidazole achieved by increasing the proportion of elution buffer (500 mM imidazole, 20 mM sodium phosphate, 500 mM NaCl, 1 mM DTT, pH 7.4) through the column. Fractions containing protein were collected and pooled, and the His affinity tag was cleaved using 150 µl of 210 µM TEV protease. The protein solution was dialyzed against PBS buffer (20 mM sodium phosphate, 150 mM NaCl and 1 mM DTT, pH 7.4) using a 3.5 kDa dialysis membrane at 20 °C. Dialyzed protein was passed through a 0.22 µm filter and the affinity tag was separated from the solution by passing the sample over a His-prep FF 16/10 column. Purified protein was concentrated and passed through HiLoad 26/60 Superdex 75 column equilibrated with PBS buffer. The major fraction containing E2-25K was collected, concentrated to 500 µM, and stored at −80 °C in 10–500 μL aliquots.

### NMR spectroscopy

NMR samples were prepared by adjusting the concentrations of wildtype, Q126L, and D127G E2-25K stored at −80 °C to ~600–700 µM. NMR samples consisted of ~300 µl of [U-^15^N] or [U-^13^C,^15^N] protein in PBS buffer (20 mM sodium phosphate, 150 mM NaCl and 10 mM DTT at pH 7.4) containing 20 µl of 99.9% D_2_O and 1 mM DSS. NMR experiments were recorded using a Varian Unity INOVA 600 MHz spectrometer. Typically, NMR samples were buffer exchanged every three days with PBS buffer containing 10 mM freshly dissolved DTT. Chemical shift assignments for E2-25K were accomplished using previous data^36^, and assignments deposited in the BMRB^37^. Assignment ambiguities were resolved through analysis of 3D HNCA, HN(CO)CA, HNCACB, and CBCA(CO)NH experiments^56–60^. Assignment of the E2-25K mutants D127G and Q126L were accomplished using a combination of chemical shift data from the BMRB and the HNCA/HN(CO)CA experiments. Main chain amide ^15^N-*R*_1_, *R*_2_, and ^1^H-^15^N NOE relaxation experiments were recorded as described previously^61^. The overall correlation times for molecular tumbling, or τ_c_ values, for E2-25K and the D127G/Q126L mutants were determined as previously described^62,63^. The impact of the D127G/Q126L mutations on the structure of E2-25K was assessed by determining main chain ^15^N and ^1^H^N^ chemical shift differences (Δδ) in 2D ^15^N-HSQC NMR spectra according to:

1 $${\rm{\Delta }}\delta =\sqrt{{({\rm{\Delta }}{\delta }^{1}{{\rm{H}}}^{{\rm{N}}})}^{2}+{({\rm{\Delta }}{\delta }^{15}{\rm{N}}/5)}^{2}}$$

where Δδ^1^H^N^ and Δδ^15^N are chemical shift differences of ^1^H^N^ and ^15^N dimension in ppm^64^. NMR spectra were processed using the NMRpipe program^65^, and chemical shift assignments were accomplished using CARA^66^. Analyses of relaxation and chemical shift perturbation experiments were done using CCPNMR^67^. Secondary structures for wildtype, Q126L, D127G E2-25K were calculated from chemical shifts using the CSI 3.0 program^68^.

### NMR monitored titration and lineshape analysis for the E2-25K–Ub interaction

A stock solution of ~800 μM [U-^15^N]-E2-25K was prepared in PBS buffer as described above. Lyophilized human ubiquitin was purchased from BostonBiochem and dissolved in identical PBS buffer as E2-25K to yield a stock concentration of ~2 mM. The concentrations of both proteins were calculated using the bicinchoninic acid assay^69^, and verified with amino acid analyses. Six separate E2-25K samples were prepared from the same stock solution for the titration, having increasing concentrations of ubiquitin of 0, 200, 400, 560, 790, and 1600 µM. The corresponding concentrations of E2-25K were 417, 420, 421, 424, 424, and 428 µM. All 2D ^15^N-HSQC NMR experiments were recorded using 32 scans and 128 increments in the ^15^N dimension. The combined change in ^1^H^N^ and ^15^N chemical shifts (Δδ), for each residue upon binding of ubiquitin to E2-25K were calculated according to Eq. 1. Δδ values were fit to a 1:1 binding isotherm to determine the dissociation constant (*K*_*D*_) as described previously^70^. Lineshape analysis was conducted to determine the kinetics of binding (*k*_*on*_ and *k*_*off*_ rates). Distinctly separated peaks in the ^15^N dimension, from the various spectra acquired for the titration, were fit using the Bloch-McConnell equations for two site exchange, as previously described^71^.

### E1 conjugation assays

E2-25K samples stored at −80 °C were thawed, freshly reduced using 10 mM DTT, and kept at 20 °C overnight. The protein solution was dialyzed against 50 mM HEPES buffer containing 150 mM NaCl at pH 8.0. The concentration of E2-25K stock solution was adjusted to 260 µM for all enzymatic assays. The ability of E1 to conjugate ubiquitin to E2-25K was quantified using reaction mixtures containing 35 mM BTP (BIS-TRIS propane), 330 nM E1, 3 mM MgCl_2_, 8 µM E2-25K or D127G and Q126L mutants, 8 µM ubiquitin labeled at the N-terminus with AlexaFluor488, and 3 mM ATP. The reaction was allowed to proceed for 90 minutes, with samples collected at various time points, whose spacing was determined depending on the rate of conjugation. E2-25K ~ Ub thioester conjugate was separated using SDS-PAGE, and quantitative analysis was accomplished using a Typhoon 9400 imager to detect AlexaFluor488 fluorescence at 517 nm. The observed rate of thioester buildup (*k*_*obs*_) was determined using a two-parameter exponential fit. The apparent rate constant (*k*_*app*_), was determined by dividing *k*_*obs*_ by the E1 concentration to facilitate rate comparisons for the different mutants.

### Hydrolysis Assays

The reaction mixtures for hydrolysis assays were similar to E1 conjugation assays. Hydrolysis was initiated by inhibiting the E1 catalyzed E2-25K ~ Ub thioester conjugation reaction with 50 mM PYR-41, an E1-specific inhibitor, and waiting 30 minutes to achieve complete inhibition^72^. Samples were collected at 20 min intervals, with quantification of E2-25K ~ Ub thioester hydrolysis achieved using SDS-PAGE with imaging of AlexaFluor-488 fluorescence as described above. The rates of thioester loss and concomitant build-up of Ub as a result of hydrolysis were calculated through fits to a two-parameter exponential decay for a first order reaction.

### Aminolysis assays

Reaction mixtures for aminolysis were similar to those for E1 conjugation, as described above. Following conjugation of fluorescent Ub to E2-25K by E1, the reaction was inhibited by PYR-41, and various concentrations of lysine, ranging from 10 to 75 mM, were added to the reaction mixture. Samples were collected at regular time intervals, and SDS-PAGE was used to separate E2 ~ Ub and Ub. Fluorescence intensities for E2 ~ Ub and Ub were fit to two parameter exponential decay and growth, to determine the rates of thioester loss and Ub build up, respectively. As previously described, the observed aminolysis rates were quadratic with respect to increasing lysine concentration, indicating that the rate law is given by^26,73^

2 $$\frac{d[E2\sim Ub]}{dt}=-\,{k}_{cat,1}[E2\sim Ub][ly{s}^{0}]-{k}_{cat,2}[E2\sim Ub][ly{s}^{0}][ly{s}^{0}]$$

where *lys*^0^ is concentration of lysine with neutral side chain at under the conditions of the reaction mixture. Plots of *k*_*obs*_/[*lys*^0^] as a function of [*lys*^0^] yield straight lines that are fit to obtain the rate constants *k*_*cat,1*_ and *k*_*cat,2*_.

### Ubiquitination assays

Reaction mixtures were similar to those for E1 conjugation assays. Formation of thioester was inhibited after ~30 minutes by addition of PYR-41, and K48-Ub_2_ synthesis was initiated by addition of 100 µM Ub lacking a fluorescent tag. K48-Ub_2_ synthesis was monitored by collecting aliquots at regular time intervals, separating the reaction mixtures using SDS-PAGE, followed by visualization and quantification of the gels by fluorescent imaging. To determine the catalytic rate constant for K48-Ub_2_ chain formation by E2-25K from these assays, the rate law corresponding to hydrolysis of thioester, ubiquitin binding to the UBA domain, and formation of K48-Ub_2_ was derived (Eq. 3):

3 $$\begin{array}{rcl}\frac{d[E2]}{dt} & = & {k}_{{H}_{2}O}[E2 \sim Ub]-{k}_{on}[E2][Ub]\\  &  & +\,{k}_{off}[E2\cdot Ub]+{k}_{U{b}_{2}}[Ub\cdot E2 \sim Ub]\\ \frac{d[E2 \sim Ub]}{dt} & = & {k}_{off}[Ub\cdot E2 \sim Ub]-{k}_{on}[E2 \sim Ub][Ub]\\  &  & -\,{k}_{{H}_{2}O}[E2 \sim Ub]\\ \frac{d[Ub\cdot E2 \sim Ub]}{dt} & = & -\,{k}_{off}[Ub\cdot E2 \sim Ub]+{k}_{on}[E2 \sim Ub][Ub]\\  &  & -\,{k}_{U{b}_{2}}[Ub\cdot E2 \sim Ub]-{k}_{{H}_{2}O}[Ub\cdot E2 \sim Ub]\end{array}$$

$$\begin{array}{rcl}\frac{d[E2\cdot Ub]}{dt} & = & -\,{k}_{off}[Ub\cdot E2]+{k}_{on}[E2][Ub]+{k}_{{H}_{2}O}[Ub\cdot E2 \sim Ub]\\ \frac{d[Ub]}{dt} & = & {k}_{off}[Ub\cdot E2 \sim Ub]+{k}_{off}[Ub\cdot E2]-{k}_{on}[E2 \sim Ub][Ub]\\  &  & -\,{k}_{on}[E2][Ub]+{k}_{{H}_{2}O}[E2 \sim Ub]+{k}_{{H}_{2}O}[Ub\cdot E2 \sim Ub]\\ \frac{d[U{b}_{2}]}{dt} & = & {k}_{U{b}_{2}}[Ub\cdot E2 \sim Ub]\end{array}$$

where the kinetics of Ub binding to the UBA domain are given by the on and off-rates (*k*_*on*_, *k*_*off*_). Hydrolysis of the E2 ~ Ub thioester is given by the rate constant ${k}_{{H}_{2}O}$, and the catalytic rate constant for K48-Ub_2_ formation given by *k*_Ub2_. The catalytic rate constant *k*_Ub2_ was obtained from numerical integration of the coupled differential equations (eq. 3), followed by numerical optimization of *k*_Ub2_ (Fig. 7b). The rate constants *k*_*on*_ and *k*_*off*_, describing the reversible binding of “acceptor” Ub to UBA domain, determined using ^15^N NMR lineshape analysis, were used for the numerical optimization. The on and off rates of Ub binding to the UBA domain of E2-25K thioester were assumed to be similar to those for unconjugated E2-25K.

### Molecular dynamics simulations for E2-25K and the Q126L/D127G active site gate mutants

A starting model for E2-25K was derived from the crystallographically determined structure (3K9O). All simulations were performed using the *ff*99SBNMR force field within the AMBER 11 or 12 suite of biomolecular simulation programs^74^. Structural models for the mutants were generated from the crystal structure (3K9O) using the mutagenesis protocol within the Pymol program. Starting models were then solvated within an octahedral box with a minimum of 12 Å between protein atoms and the box edges using the TIP3P water model. Na^+^ ions were added to balance the net negative charge on E2-25K and ensure system neutrality. The steepest descent method was used for initial energy minimization followed by conjugate gradient minimization. Bonds involving hydrogen atoms were constrained using the SHAKE algorithm. The system temperature was managed using Langevin dynamics with a collision frequency of 1 ps^−1^. Pairwise non-bonded and electrostatic interactions were calculated using a particle mesh Ewald approach and a distance cutoff of 8 Å. The system was heated from 0 to 298 K over 50 ps with 0.1 kcal/mol restraints applied to all the solute atoms, followed by equilibration to 1 atm pressure for 50 ps. Typically two to five ~400–800 ns production dynamic runs were conducted for each of the D127G/Q126L mutants and wildtype E2-25K. The total simulation times for wildtype, D127G, and Q126L E2-25K were 3.56, 2.83, and 1.62 μs, respectively.

Time dependent fluctuations of the active site gate were determined from the MD simulations by calculating the distance between the C_α_ atoms of the active site C92 and Q126 (or Q126L) from the active site loop over the duration of the simulation. For distances exceeded 12 Å, the gate was considered to be in an open conformation, otherwise the gate was considered to be in a closed conformation. The rates of opening and closing were determined by dividing the number of open or closed transitions by the total time in the open or closed states, respectively. Simulations for E2-25K with covalently attached ubiquitin were initially set up as described previously^26,75^. C_α_–C_α_ restraints and a restraint between E2-25K N83 HD22 and Ub G76 O were used during equilibration of the structure, after which the restraints were gradually relaxed over 4 ns. Two to three runs of ~400–800 ns for each of WT, Q126L, and D127G thioester-bound E2-25K were performed, for a total of 2.19, 1.59, and 2.34 μs, respectively.

Steered MD simulations were performed in AMBER to investigate the energetic barrier to the opening of the active site gate^76^. WT, Q126L, and D127G E2-25K systems were generated and equilibrated as described above, then equilibrated for a further 100 ps with a 10 kcal/mol/Å^2^ restraint between the Q/L126 C_α_ in the gate and the center of protein, as defined by the average position of the C_α_ atoms of E2-25K but not including the gate (residues 122–128). After equilibration, the restraint distance was increased at a speed of 0.3 Å/ns, and the work performed on the system by pulling the restraint was monitored as a function of the restraint distance. A total of 150 runs for each protein were performed, and the barrier heights were calculated using the cumulant expansion method^77^.

## Electronic supplementary material

Supplementary Information
