# Supplementary material for: Active Site Gate Dynamics Modulate the Catalytic Activity of the Ubiquitination Enzyme E2-25K
Source: Sci Rep. 2018 May 3;8:7002. doi: 10.1038/s41598-018-25476-8 (PMC5934386; doi:10.1038/s41598-018-25476-8)
Supplement: Supplementary file 1 — Supplementary Information [file 41598_2018_25476_MOESM1_ESM.pdf]

Supplementary Information

Active Site Gate Dynamics Modulate the Catalytic Activity of the Ubiquitination Enzyme E2-25K

Manoj K. Rout†, Brian L. Lee†, Aiyang Lin‡, Wei Xiao‡, and Leo Spyracopoulos†,*

†Department of Biochemistry, Faculty of Medicine and Dentistry, University of Alberta, Edmonton, Alberta, Canada T6G 2H7.

‡Department of Microbiology and Immunology, University of Saskatchewan, Saskatoon, Saskatchewan, S7N 5E5, Canada.

*To whom correspondence should be addressed

e-mail: leo.spyracopoulos@ualberta.ca.

phone: (780) 492-2417

fax: (780) 492-0886

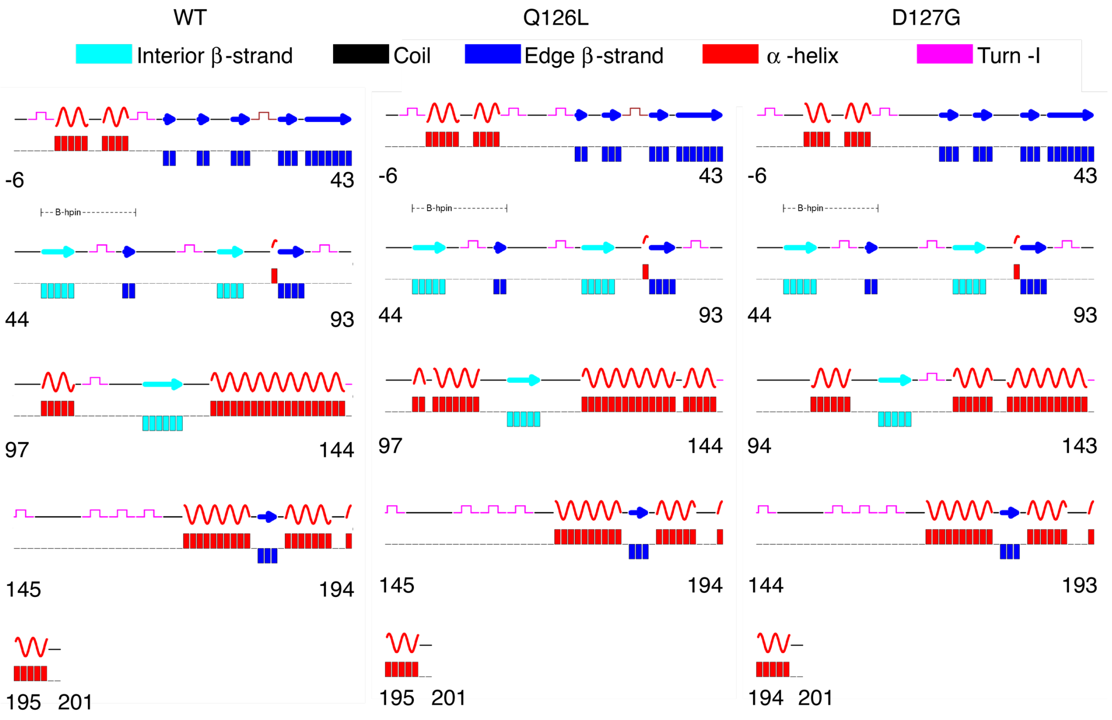

Supplementary Figure S1: Predicted secondary structures calculated using ^{15}N, $^{1}H^{N}$, CO and CA chemical shifts for wildtype, Q126L, and D127G E2-25K. The overall secondary structures of both the proteins remain similar to wildtype.

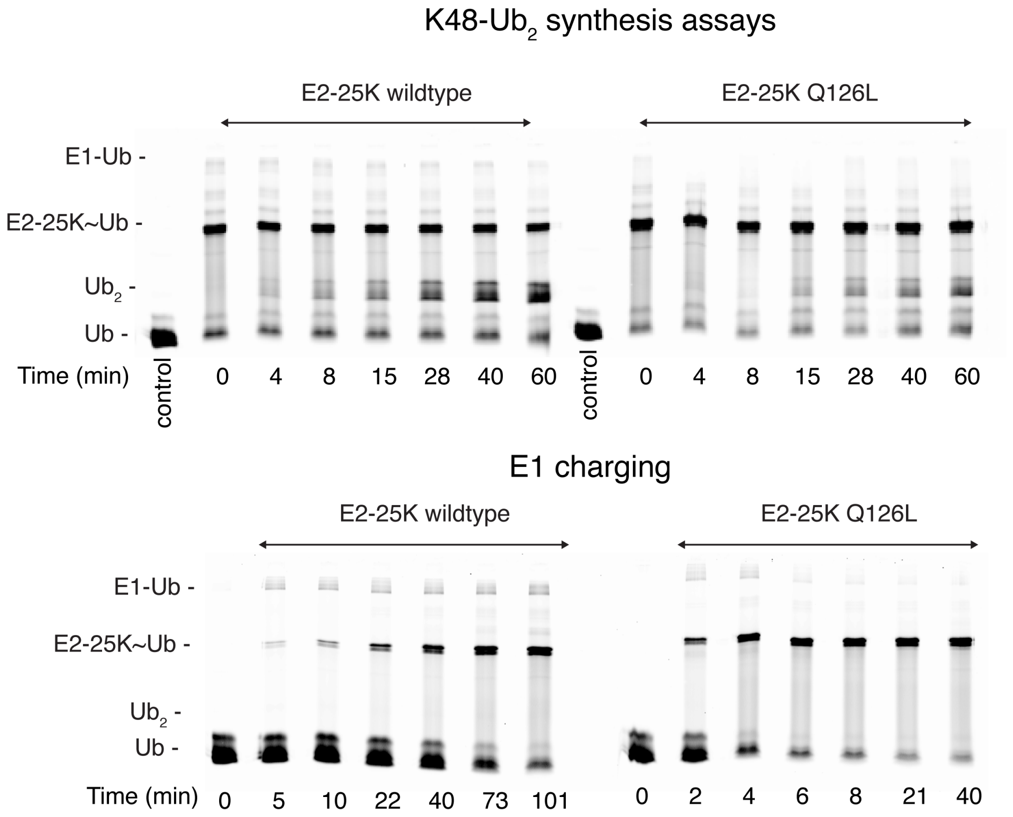

Supplementary Figure S2: Representative SDS-PAGE results for E2-25K enzyme assays for catalysis of the synthesis K48-Ub_2 chains (top) and E1-catalyzed charging of E2-25K to form a thioester with the C-terminus of Ub (bottom). Data for wildtype enzyme and the Q126L mutant are shown.

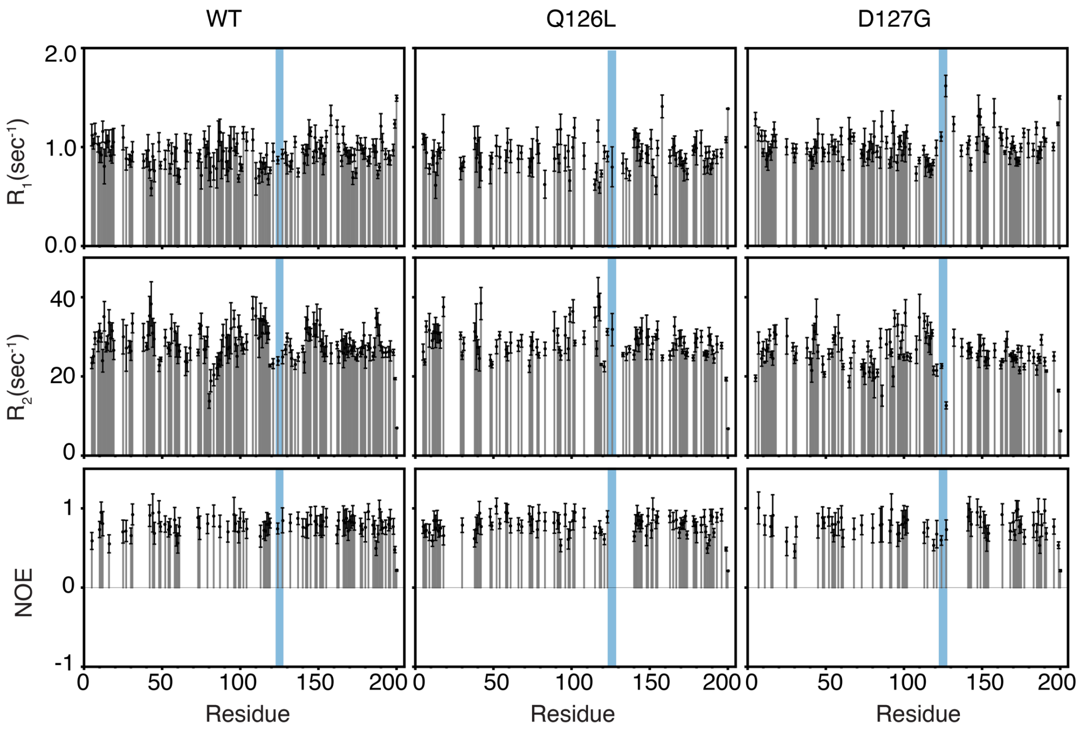

Supplementary Figure S3. 600 MHz backbone ^{15}N relaxation data for E2-25K wildtype, Q126L and D127G. Residues from 123-127 from the active site loop are highlighted with a blue bar.

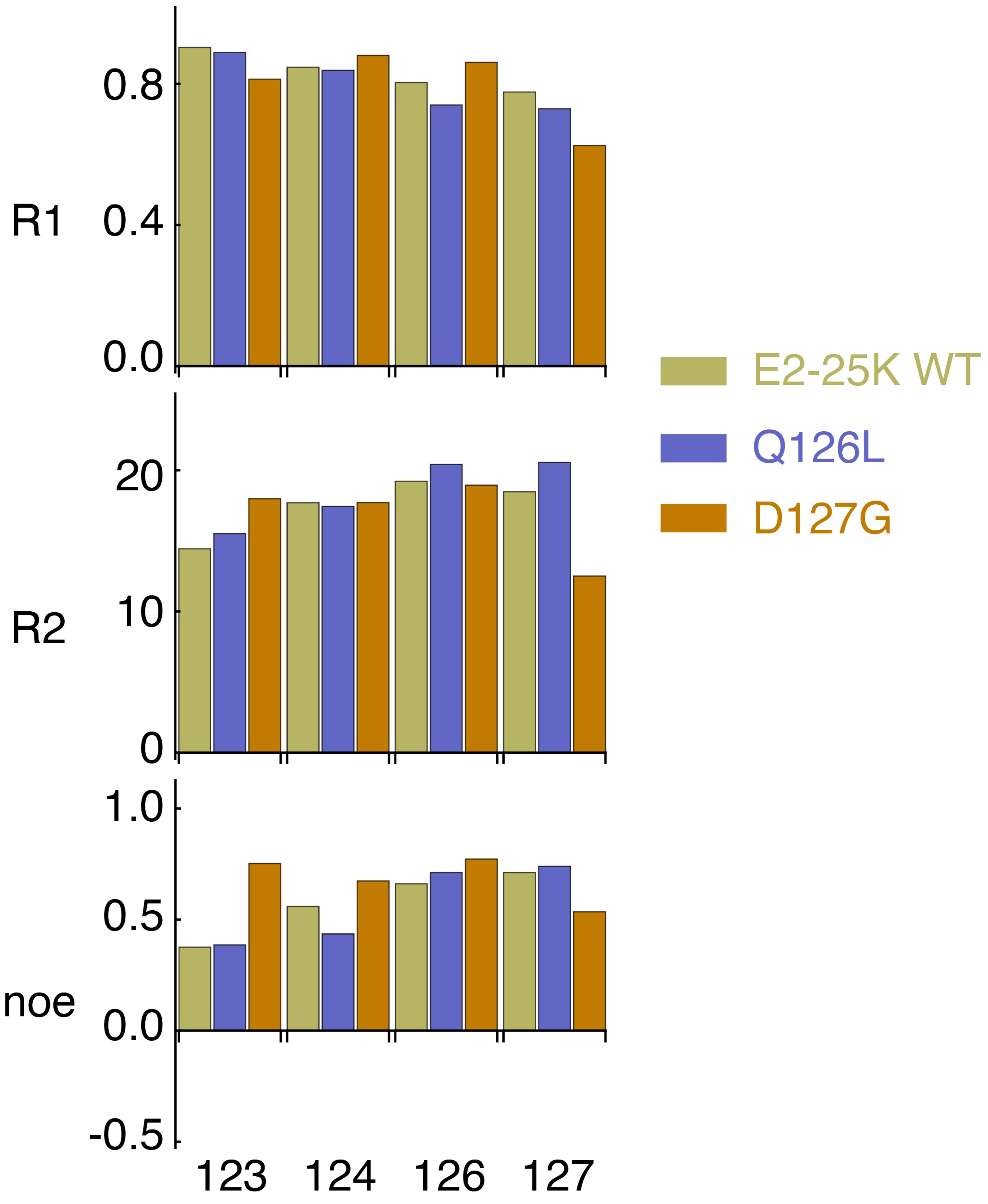

Supplementary Figure S4. MD-derived 600 MHz backbone ^{15}N relaxation data for E2-25K wildtype, Q126L and D127G.

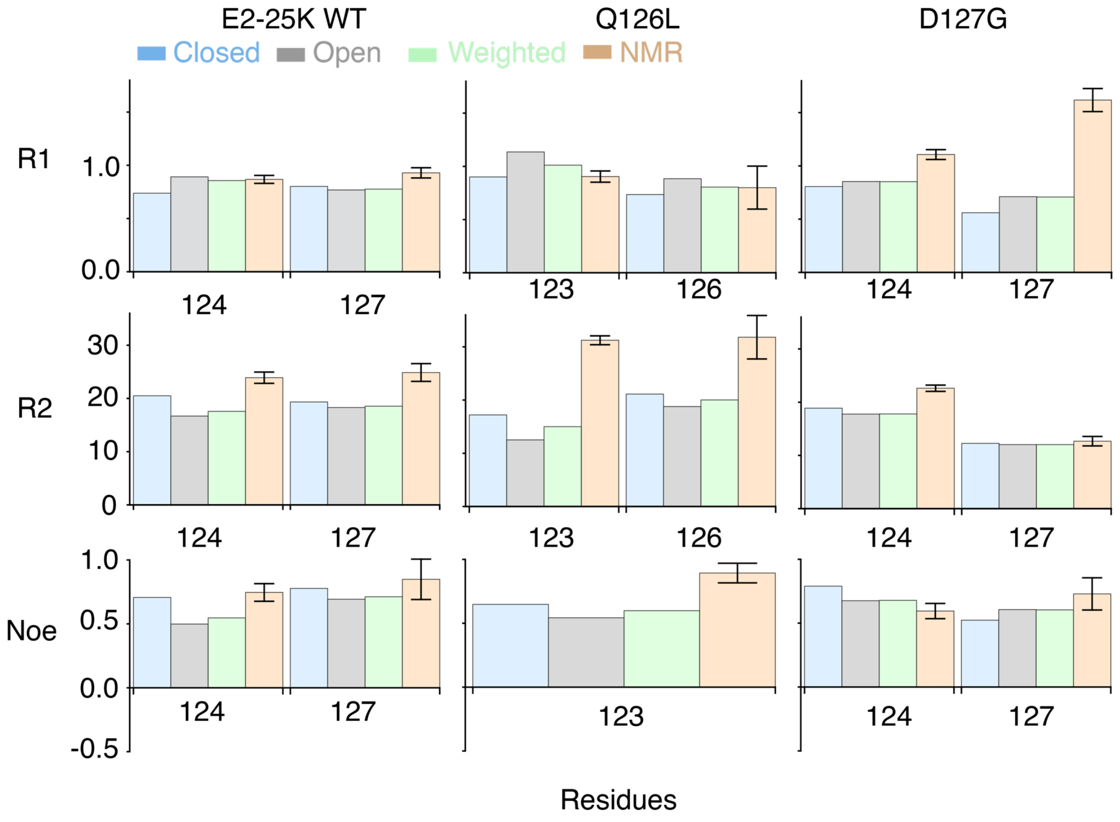

Supplementary Figure S5. Comparison of experimental and calculated 600 MHz backbone ^{15}N relaxation data for E2-25K wildtype, Q126L and D127G. The active site loop encompasses residues 123-127.
